# Biphasic Dose–Response Induced by PCB150 and PCB180 in HeLa Cells and Potential Molecular Mechanisms

**DOI:** 10.1177/1559325820910040

**Published:** 2020-03-16

**Authors:** Ainy Zehra, Muhammad Zaffar Hashmi, Abdul Majid Khan, Tariq Malik, Zaigham Abbas

**Affiliations:** 1 Department of Zoology, University of Punjab, Lahore, Pakistan; 2 Department of Chemistry, COMSATS University Islamabad, Pakistan; 3 Department of Pharmacy, Islamia University Bahawalpur, Pakistan; 4 Ministry of Climate Change, Islamabad, Pakistan

**Keywords:** hormesis, PCBs, HeLa cells, oxidative stress, antioxidant enzymes, MAPKs

## Abstract

The polychlorinated biphenyls (PCBs) are persistent and their dose-dependent toxicities studies are not well-established. In this study, cytotoxic and genotoxic effects of PCB150 and PCB180 in HeLa cells were studied. The 3-(4,5-dimethylthiazol-2-yl)-2,5-diphenyltetrazolium bromide (MTT) assay indicated that the cell proliferation was stimulated at low doses (10^−3^ and 10^−2^ µg/mL for 12, 24, 48, and 72 hours) and inhibited at high doses (10 and 15 µg/mL for 24, 48, and 72 hours) for both PCBs. Increase in reactive oxygen species formation was observed in the HeLa cells in a time- and dose-dependent manner. Malondialdehyde and superoxide dismutase showed increased levels at high concentrations of PCBs over the time. Glutathione peroxidase expression was downregulated after PCBs exposure, suggested that both PCB congeners may attributable to cytotoxicity. Comet assay elicited a significant increase in genotoxicity at high concentrations of PCBs as compared to low concentrations indicating genotoxic effects. PCB150 and PCB180 showed decrease in the activity of extracellular signal–regulated kinase 1/2 and c-Jun N-terminal kinase at high concentrations after 12 and 48 hours. These findings may contribute to understanding the mechanism of PCBs-induced toxicity, thereby improving the risk assessment of toxic compounds in humans.

## Introduction

Polychlorinated biphenyls (PCBs) are ubiquitous organochlorine pollutants elicit different chemical and toxicological properties. Polychlorinated biphenyls are categorized according to their degree of chlorination (low- and high-chlorinated PCBs), substitution pattern (chlorine substituent in ortho-, meta-, para-position), or by producing similar effects as other chemicals such as dioxin-like (DL) PCBs within biological systems.^1^ Polychlorinated biphenyls characteristics (inflammability and electric conductivity) have brought them to a range of applications such as coolants, flame retardants, and hydraulic fluids, and (pronounced lipophilicity, persistence, and degradability) enable them to be readily deposited in human, fish, bird, and plant tissues.^2^ Polychlorinated biphenyls were introduced in the late 1920s, used until the 1970s and finally internationally banned in 1979. Polychlorinated biphenyls are included in the Stockholm Convention on Persistent Organic Pollutants, aims to eliminate emissions of persistent organic pollutants.^3^ They have been classified into nonorthochlorinated or DL and orthochlorinated or non-dioxin-like (NDL) groups which have a coplanar and noncoplanar spatial structure, respectively. The toxic potency of each group is structure-dependent and usually based on chlorine position. Both groups are considered to contribute substantially to severe toxic consequences and cumulative adverse health effects including risk of cancer, immune suppression, endocrine disruption, metabolic dysfunction, genotoxic effects, and reproductive and nervous system abnormalities.^4^

In general, cells can respond to damage and stress by activating various repair and survival pathways.^5^ This process involves low-level exposure of an environmental factor chemical (heavy metals, trace elements insecticides, pesticides, etc), physical (electrical, mechanical, heat, cold, etc), and biological (bacterial, viral, etc) induces beneficial effects and toxic effects at high-level exposure in the cell or organism.^6^ Biphasic effects can be induced by various stimuli (radiation, toxins, natural compounds, pharmaceutical agents and endogenous agonists) in many biological models (microbes, plants, invertebrates, and mammals) suggesting that it is independent of biological model, end point measured, chemical class, and interindividual variability.^7^ Hormetic responses are typically plotted on graphs as either inverted U-shaped or J-shaped dose–responses that contact the control value at 2 points.^8^ A significant feature of the hormesis concept is its consistent quantitative features including the magnitude (30%-60% more than the control response), width of stimulation (within 100-fold of the zero-equivalent point), and their respective relationship to the zero-equivalent point (ie, threshold).^9^ Moreover, it mediates cellular stress in a broad range of preventive, reparative, and signaling activities and regarded as a set of evolutionarily conserved adaptive mechanism.^10^

Aside from these, several studies revealed that exposure to PCBs can lead to severe toxic consequences and adverse health effects such as modulation of the intracellular calcium homeostasis,^11^ altered thyroid hormone homeostasis,^12^ altered neurotransmitter signaling,^13^ induced reactive oxygen species (ROS),^14^ altered cell viability,^15^ alterations of neuro/endocrine processes,^16^ and membrane disruption.^17^ Of the modes identified, the ability of PCBs to enhance cellular oxidative stress is potentially of greater concern. However, a detailed knowledge of the mechanisms involved in the hormetic approach enable to simulate levels of the PCB congeners is needed in order to identify the possible mechanisms of toxicity in HeLa cells. These cells were derived from a glandular cervical cancer and widely referenced approximately more than 100 000 publications of biological studies.^18^ These cells are particularly robust and fast-growing and consequently can rapidly overgrow than other cells^19^ and sensitive to chemical toxicity.^20^ These cell lines have also been explored for biological studies for tumor cells as well as their biphasic dose–response have also been evaluated for various compounds including 1-hexadecyl-3-methylimidazolium chloride^21^ and digitalis compounds.^22^ Hence, HeLa cells are hardly studied yet and scarcity of data in respect to the toxic effects of PCBs and their involved mechanisms.

Using biphasic dose–response model, the present study use HeLa cells to analyze the effects of 2 different PCB congeners: 2,2′,3,4′,6,6′-Hexachlorobiphenyl (PCB150) and 2,2′,3,4,4′,5,5′-Heptachlorobiphenyl (PCB180) as the test compounds. As known, both congeners exhibit different behavior in the environment. However, PCB150 is yet poorly characterized in terms of its toxicological effects, and PCB180 is the most prevalent in the environment and biota and frequently used to monitor in animals and humans.^23^ Furthermore, it shows pronounced accumulation in food chains and is found at elevated levels in biological samples rapidly.^24^ To this aim, this study provides mechanistic insights into how exposure assessment of PCB150 and PCB180 in HeLa cells evokes oxidative stress that modifies the cellular redox state, parameters of molecular defense mechanism involving cell proliferation, activity of oxidant and antioxidant enzymes, and activation of mitogen-activated protein kinases (MAPKs) signaling pathways (extracellular signal–regulated kinases [ERKs] and JNKs) to determine how each factor affects one another to respond in oxidative stress condition, leading to their survivorship.

## Materials and Methods

### Chemicals and Reagents

PCB150 and PCB180 were purchased from Accustandard, Inc (New Haven, Connecticut), Dulbecco’s Modified Eagle’s Medium (DMEM) and Fetal Bovine Serum (FBS) from GIBCO BRL and Life Technologies (Grand Island, New York), The 2 dichlorofluorescein diacetate (DCFH-DA), 3-(4,5-dimethylthiazol-2-yl)-2,5-diphenyltetrazo-lium bromide (MTT), and dimethyl sulfoxide (DMSO) from Sigma Chemical (St Louis, Missouri). HeLa cells from Shanghai Institute of Cell Biology, Chinese Academy of Sciences (Shanghai, China), the detection kits for malondialdehyde (MDA) content, Glutathione peroxidase (GSH-Px) activity, and superoxide dismutase (SOD) activity from Nanjing Jiancheng Biological (Jiancheng, New Jersey). All other reagents used were of analytical grade or highest grade available. A 20 µg/mL stock solution of PCB was prepared in DMSO and diluted further to working concentrations. The final concentration of DMSO in the culture medium was lower than 0.2% (vol/vol).

### Cell Culture

HeLa cell was cultured in DMEM, supplemented with 10% (vol/vol) fetal calf serum and penicillin–streptomycin (100 IU/mL-100 µg/mL) in a humidified incubator at 37°C with 5% CO_2_.

### 3-(4,5-dimethylthiazol-2-yl)-2,5-diphenyltetrazolium bromide Assay

The MTT assay is measuring of survival and/or proliferation of cells and was used as indicator of cell viability by determining the amount of formazan crystals produced by metabolic activity in treated versus untreated control cells. Exponentially growing cells (1 × 10^4^/well) were seeded in 96-well plates for 24 hours. Cell media with different concentrations of PCB150 and PCB180 were added for 12, 24, 48, and 72 hours, respectively. After that, cells were incubated for 4 hours with MTT solution (0.5 mg/mL) at 37°C in humidified incubator with 5% CO_2_. The medium was then aspirated, and DMSO was used to dissolve the crystals. Absorbance was recorded at 570 nm using SpectraMax Plus 384 microplate reader (Sunnyvale, California).

### Reactive Oxygen Species Detection

Reactive oxygen species levels were measured, and the probe DCFH-DA was dissolved in DMSO as a 50 mM stock. Cells were washed and scraped in phosphate-buffered saline (PBS) at a density of 1 × 10^5^ cells/mL as 1-mL cultures. Cultures were incubated with 100 µM DCFH-DA for 15 minutes after different concentrations of PCB150 and PCB180 with control, 10^−3^, 10^−2^, 1, 10, 15 µg/mL for 12, 24, 48, and 72 hours. All samples were incubated at 37°C for 1 hour and immediately analyzed by SpectraMax Plus 384 microplate reader using 504 nm excitation.

### Assay of Antioxidant Enzymes

The activities of antioxidant enzymes in PCB150 and PCB180 treated HeLa cells were measured, as described by Jiancheng Biochemical (Nanjing, China) Assay Kit.

### Determination of MDA Content

For MDA, the cell suspension extract (0.5 mL) was put into a test tube and was added by 2-mM sodium azide in 4.5 mL of 0.1M PBS (pH 7.4). The mixture was incubated at 37°C for 1 hour, after that 2 mL of 28% wt/vol trichloroacetic acid was added. The tube was vortexed, cell suspension was centrifuged at 1000 3 g for 5 minutes and then 4 mL supernatant was transferred to a tube to which 1 mL of 1% wt/vol thiobarbituric acid was added. It was bathed in boiling water for 15 minutes and after cooling the absorbance was read at 532 nm using a SpectraMax Plus 384 microplate reader.

### Determination of GSH-Px Content

For GSH-Px, the cell suspension extract (100 µL) was mixed with 700 µL reaction mixture included 1 mM ethylenediaminetetraacetic acid (EDTA), 1 mM sodium azide, 0.2 mM NADPH, and 1 mM glutathione in PBS (pH 7.2) and 100 µL containing 10 U glutathione reductase. The tubes were vortexed and incubated for 5 minutes at room temperature. After incubation, the absorbance was recorded at 340 nm immediately using a SpectraMax Plus 384 microplate reader.

### Determination of SOD Content

For SOD, the cell suspension extract (200 µL) was mixed with 750 µL Tris cacodylic buffer, included 50 mM Tris-HCl, 50 mM cacodylic acid, and 1 mM EDTA (pH 8.2), followed by the addition of 250 µL of 2 mM pyrogallol. Absorbance was determined immediately at 550 nm using a SpectraMax Plus 384 microplate reader.

### Protein Assay

Bradford (1976) assay was performed in order to measure cell protein in HeLa cells and immediately absorbance was recorded at 595 nm using a SpectraMax Plus 384 microplate reader.

### Comet Assay

By following the method of Tice et al,^25^ the comet assay was determined in PCB150- and PCB180-treated HeLa cells. Normal melting agarose (NMA) and low-melting agarose (LMA) solutions were prepared in PBS. Sequentially, 100 µL 1% NMA was coated on warm frosted comet slide. Then, cells after exposure were suspended in 0.7% LMA, and 75 µL of this solution which was pipetted onto the first layer of gel. Finally, the third layer (80 µL of 0.5% LMA) was added. The precipitated “sandwich” gel was incubated in Lysis solution (2.5M NaCl, 100 mM Na2EDTA, 10 mM Tris, 1% Triton X, 10% DMSO, pH 10.0) and placed at 4°C for 1 hour and then electrophoresed in cooled alkaline electrophoresis buffer (300 mM NaOH, 1 mM Na2EDTA, pH 12.5) for 20 minutes at 25 V and 300 mA. Following electrophoresis, slides were incubated with neutralization buffer (0.4M Trizma base, pH 7.5, 4°C) for 8 minutes twice and air dried. The slides were stained with PI (20 µg/mL) and evaluated under a fluorescence microscope. The extent of the genotoxic effect of PCBs was calculated using the CASP software (University of Wroclaw, Poland) with 5 comet parameters–tail length (TL; distance from nuclear center to the end of the comet tail), tail DNA percentage (TD% [expressed by the percent of fluorescent intensity in tail]), head DNA percentage (HD% [expressed by the percent of fluorescent intensity in head]), tail moment (TM; product of TL and TD), and comet length. All experimental steps were performed under dim red light at 4°C to avoid additional DNA damage.

### Western Assay

Cells were lysed with cell lysis buffer (300 mL of ice cold 50 mM Tris-HCl [pH 7.4], 150 mM NaCl, 50 mM NaF, 1 mM Na3VO, 5 mM NaF EDTA, 50 mMNaPPi, 1 mMPMSF, 1 mMDTT, 5 mg/mL leupeptin, 2 mg/mL aprotinin, and 1% NP-40) and lysates were harvested by centrifugation at 12 000 × g for 30 minutes at 48°C to remove the debris. Total protein amounts were obtained with polymer of vinylidene fluoride membranes (Bio-Rad Laboratories, Hercules, California). Lysates were then separated by 10% SDS-PAGE and transferred to nitrocellulose membrane (Hy-bond-ECL; Amersham Pharmacia Biotech, Buckinghamshire, United Kingdom) and blocked with 5%, wt/vol, nonfat dry milk. The primary antibodies (1:1000) used include c-Jun amino-terminal kinase (JNK), ERK, as p-JNK, ERK1/2, p-ERK1/2, and actin. Then, sequentially incubated with peroxidase-conjugated secondary antibody (1:5000) for 2 hours in ambient conditions. To confirm even loading, membranes were stripped and probed with b-actin antibody (Cell Signaling Technology, Beverly, Massachusetts). Visualization was performed with the optical densitometry using the software ImageJ (NIH) to quantify the intensities of the immunoreactive bands. The expression of actin was shown as loading control. Results are representative of 3 independent experiments.

### Statistical Analysis

Statistical analysis was done by SPSS version 18.0 (SPSS, Chicago, Illinois). Results were expressed as means ± standard deviation. Analysis of variance was done with the least significant difference to compare means of different PCB150 and PCB180 concentrations with control. For all data sets 2 tailed, unpaired Student *t* tests were performed, and a value of *P* < .05 was considered statistically significant.

## Results

### HeLa Cell Proliferation

**Figure 1.**
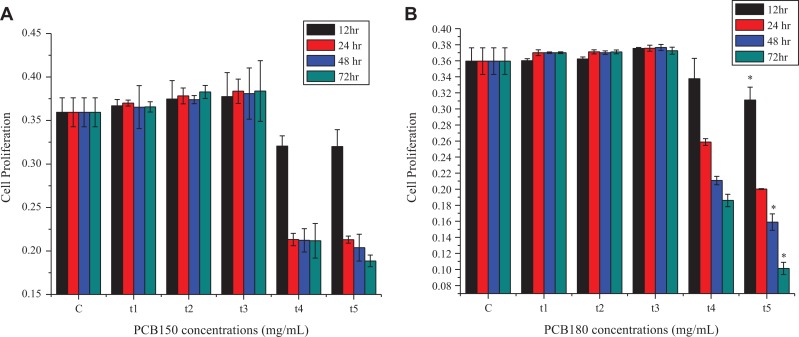
(A) Effects of PCB150 (B) PCB180 on HeLa cell proliferation under different concentrations (Control [C], t1 [0.001], t2 [0.01], t3 [1], t4 [10], t5 [15] µmg/mL) for (12, 24, 48, and 72 hours). PCB indicates polychlorinated biphenyl.

Using MTT proliferation assay as described in methods, we evaluated the dose–response of PCB150 and PCB180 in HeLa cells (Figure 1A and B). To investigate the cell proliferation, the subsequent experiment was designed, and cells were administered with various concentration of PCBs 10^−3^, 10^−2^, 1, 10, and 15 µg/mL for 12, 24, 48, and 72 hours. The results revealed that the HeLa cells exhibited biphasic dose–response in concentrations and time-dependent manner. Experimental variations enable us to suggest that PCB150 exposed HeLa cells were slightly proliferated after treatment with 10^−3^ and 10^−2^ µg/mL over time and did not show cytotoxicity up to the concentration of 1 µg/mL. In contrast, treatment with 10 and 15 µg/mL for 24, 48, and preferably 72 hours substantially reduced cell proliferation. In addition to this, PCB180 indicated a marked decrease in cell proliferation after treatment with 10 and 15 µg/mL for different time. The comparison between 2 congeners (PCB150 and PCB180) illustrated maximum stimulatory response at 10^−3^, 10^−2^, and 1 µg/mL and inhibitory response at 10 and 15 µg/mL. This study indicated that this biphasic dose–response phenomenon was in line with the typical character of hormesis.

## Oxidative Stress Mediate ROS Signaling

**Figure 2.**
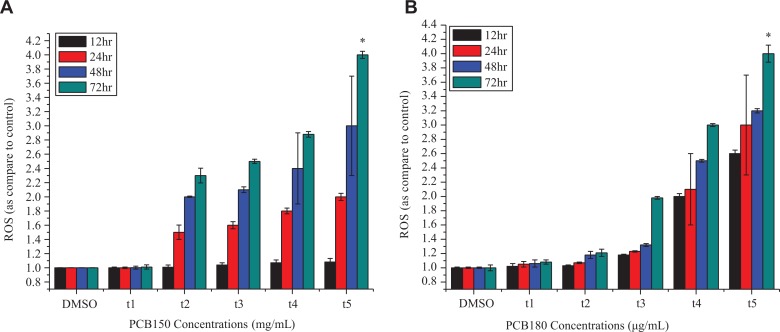
(A) Effects of PCB150 and (B) PCB180 ROS production under different concentrations and time. PCB indicates polychlorinated biphenyl; ROS, reactive oxygen species.

The results of oxidative stress in HeLa cells exposed to PCB150 and PCB180 with upstream ROS activation are given in Figure 2A and B. Among the stress signals, ROS is an elicitor of oxidative stress and is formed as natural byproduct in various intracellular metabolism.^26^ Reactive oxygen species may be also highly damaging, as they can attack biological macromolecules, namely, lipids, proteins, and DNA, induce oxidation and cause membrane damage, enzyme inactivation, and DNA damage. However, the level of ROS exceeds the antioxidant capacity of the cell, the intracellular redox homeostasis is altered, and oxidative stress ensues.^27^ Our results elicited greater toxicity by PCB150 which showed great elevated ROS generation. It was found that at low dose, 10^−3^ µg/mL of PCBs exposure for 12 and 24 hours the cells remained completely unaffected except for 48 and 72 hours. Reactive oxygen species levels increased at the concentrations of 10^−2^, 1, 10, 15 µg/mL in comparison with the control (DMSO treated) at 48 and 72 hours. On the other hand, PCB180 depicted moderate toxicity and ROS level was slightly increased after 12, 24, and 48 hours for 10^−3^, 10^−2^, and 1 µg/mL. The concentrations 10 and 15 µg/mL for 48 and 72 hours showed elevated ROS. This increase was dose- and time-dependent. Collectively, this finding suggested that PCB150 and PCB180 are capable to induce oxidative stress at high doses and downstream ROS at low doses.

### Expression Level of Antioxidant Enzymes (MDA, GSH-Px, and SOD)

**Figure 3.**
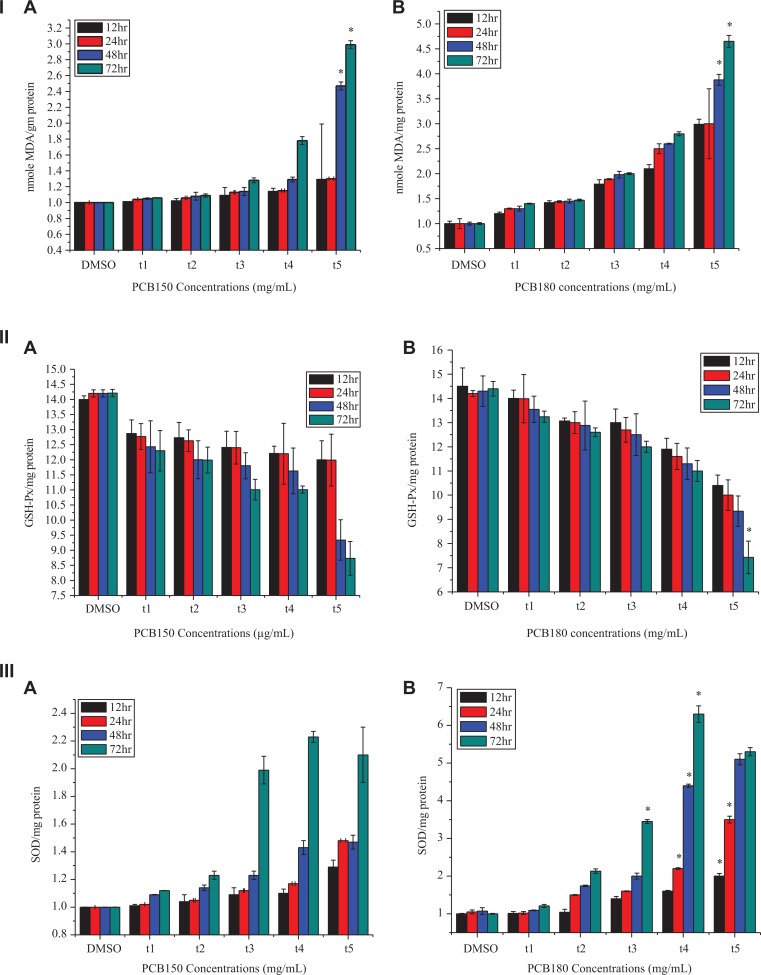
I (A) Effects of PCB150 and (B) PCB180 on MDA contents; II (A) GSH-Px contents (B) GSH-Px. III (A) SOD contents and (B) SOD contents, respectively, under different concentrations and time. MDA indicates malondialdehyde; PCB, polychlorinated biphenyl; SOD, superoxide dismutase.

The study aimed to gain insight into the defense mechanism for ROS scavenging by antioxidant enzymes. To scavenge excessive ROS, efficient enzymatic cellular antioxidant machinery includes multiple components such as SOD, glutathione peroxidase, catalase, and peroxiredoxin which convert ROS into less noxious compounds. An increased ROS scavenging capacity is positively correlated with the prevention of oxidative stress. Thus, ROS detoxification is crucial for the balance of ROS accumulation. In this study, HeLa cells were evaluated for MDA, SOD, and GSH-Px by lipid peroxidation assay (Figure 3).

The expression of MDA is the product that oxygen-free radicals react with in biological membrane unsaturated fatty acids. Its content can reflect the degree of lipid peroxidation, indirectly reflecting cell damage.^28^ The data explored that no significant change in expression of MDA after treatment with PCB150 at all concentrations for 12 and 24 hours. However, a markedly enhanced expression at 48 and 72 hours for 10 and 15 µg/mL than that of control indicated relationship between MDA and ROS in oxidative stress (Figure 3I-A). Furthermore, PCB180 induced the accumulation of MDA with rise of concentrations. The MDA expression was upregulated at 10 and 15 µg/mL at different times indicated that lipid peroxidation promoted by higher levels of dose and time (Figure 3I-B).

To further investigate the ROS detoxification, the activities of GSH-Px were observed which not only act as primary line of defense to cope with deleterious effects of ROS but also contributing to an overall decrease in oxidative damage.^29^ Our results showed that PCB150 decreased the level of GSH-Px contents at all concentrations (Figure 3II-A). Interestingly, there is remarkable decrease in accumulation of GSH-Px as compared to control after treatment with 15 µg/mL as treatment time extended. Concomitantly, the expression of GSH-Px for PCB180 was consistent with PCB150 and dramatically decrease accumulation was noticed at various time for 15 µg/mL (Figure 3II-B). In our study, the GSH-Px content suggested an obvious oxidative damage in HeLa cell.

The SOD act as the antioxidant enzyme and can protect cells from the potentially harmful effects of ROS in vitro and in vivo. To assess SOD production in HeLa cells for PCB150, the results indicated a selective increase at all concentrations at various times. However, significant exacerbation was noticed after exposure to 48 and 72 hours for 1, 10, and 15 µg/mL (Figure 3III-A). PCB180 increased the level of SOD reaching to highest level for 72 hours at all concentrations except 10^−3^ µg/mL (Figure 3III-B). The results show that the antioxidant enzymes have adaptive characteristics altering their activity to scavenge excess ROS reduces the level of mitochondrial membrane lipid peroxidation to maintain the normal physiological function in HeLa cells exposed to PCB150 and PCB180.

### Polychlorinated Biphenyls–Induced Genotoxicity

**Figure 4.**
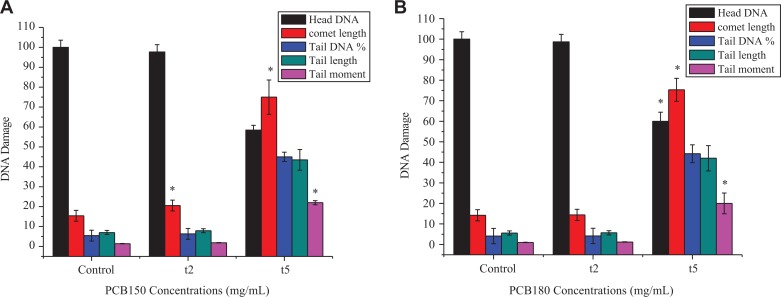
(A) Effects of PCB150 and (B) PCB180 on DNA damage under different concentrations and time. PCB indicates polychlorinated biphenyl.

The genotoxic effects of PCB150 and PCB180 upon HeLa cells are given in Figure 4. The comet assay (single cell gel electrophoresis [SCGE]) is the most widely used method for measuring DNA damage in eukaryotic cells. It has been widely used in genotoxicity testing of chemicals, in both in vitro and in vivo models. The theory governing the comet assay is that genotoxicants can induce DNA damage in the form of single-strand breaks, and alkali labile sites or adducts that convert to DNA strand breaks under alkali treatment. Fragmented DNA can more readily migrate, and single-strand breaks can release superhelical tension, allowing for loops of DNA to migrate toward a positively charged anode. The image of the migrated DNA resembles a comet, from which the assay gets its name. In this study, the results indicated that PCB150 have no significant differences after treatment of 10^−2^ µg/mL (t2) as compared to the control (Figure 4A). In the control samples, percentage of head DNA was more than 97% and the comet length, tail DNA, TL, and TM were lower than 20%. After treatment with 1 µg/mL (t3), the significant elevation of DNA damage was detected and percentage of head DNA decreased below 60%, while the comet length, tail DNA, TL, and TM significantly increased. The genotoxicity upon HeLa cells for PCB180 revealed that the results are in accordance with PCB150 after treatment with 10^−2^ µg/mL (t2). However, DNA damage induced in a concentration-dependent manner and head DNA decreased, while all the rest parameters increased significantly compared to control (Figure 4B). These results also showed that PCB150 and PCB180 are potent promoters of DNA damage especially at the highest level of exposure.

### Activation of MAPK Signaling Pathways

**Figure 5.**
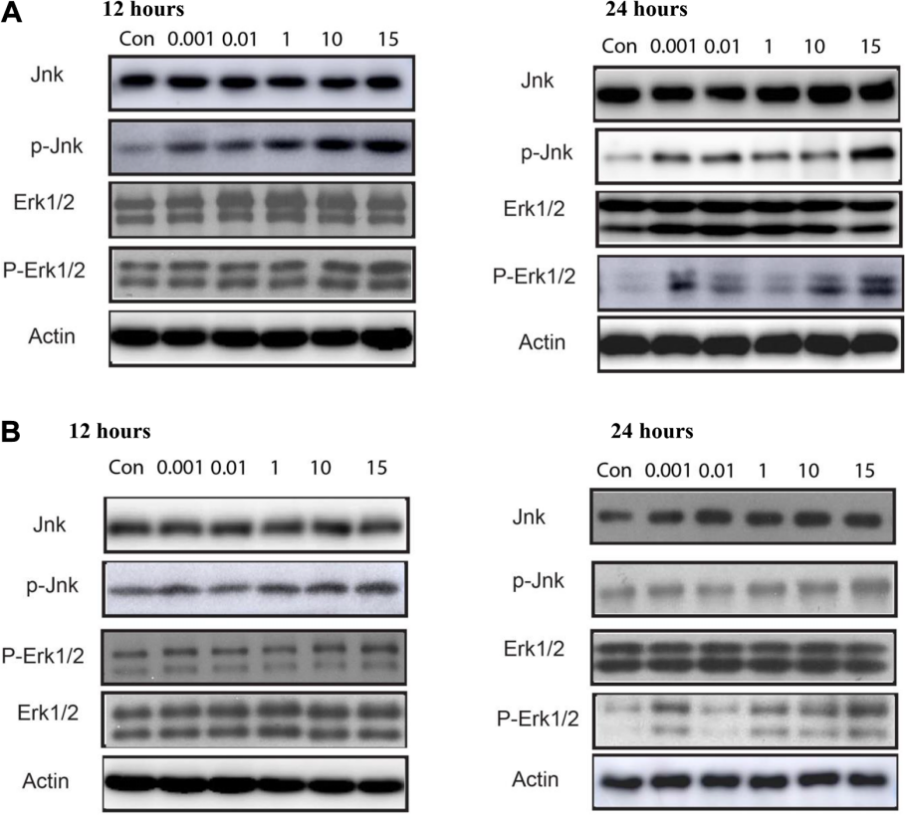
(A) Effects of PCB150 and (B) PCB180 on JNK and ERK1/2 proteins under different concentrations and time. ERK indicates extracellular signal–regulated kinase; PCB, polychlorinated biphenyl.

The phosphorylation status of JNK, p-JNK, ERK1/2, p-ERK1/2, and actin was analyzed to understand the effects of different concentration 10^−3^, 10^−2^, 1, 10, and 15 µg/mL for 12 and 48 hours of PCB150 and PCB180 (Figure 5A and B). In this study, extracellular signal transduction pathways were determined by using Western assay to ascertain whether JNK and other stress-related MAPKs, such as p-JNK, ERK1/2, p-ERK1/2, and actin-driven cell death in HeLa cells during PCBs exposure. The results indicated that JNK, p-JNK, ERK1/2, and p-ERK1/2 were downregulated at the higher concentrations of both PCBs. Following this, the relative phosphorylation of JNK, p-JNK, ERK1/2, p-ERK1/2, and actin was exposed at concentration of 10^−3^, 10^−2^ µg/mL and slightly increased at 1, 10, and 15 µg/mL for 12 hours in HeLa cells exposed to PCB150 and PCB180 indicating that the lower levels of JNK activation in HeLa cells were responsible for their resistance to autophagic cell death (Figure 5). However, the proteins elicited higher phosphorylation in dose-dependent manner when incubated for 48 hours for both congeners, suggesting an intimate coupling between alterations in the intracellular redox state and the activity of downstream stress-activated pathways (Figure 5). Consistent with previous study, PCBs induce apoptosis in several cell lines and JNK might act as a pivotal signaling molecule and sustained activation of JNK is reported to be linked with cell death. These findings imply a clear dose–response relationship for the phosphorylation of JNK cascade and therefore suggest that oxidation of PCB150 and PCB180 boosts endogenous level of apoptotic proteins pathways under oxidative stress and subsequently produces DNA damage.

## Discussion

The adaptive response of living system to environmental stress is commonly known as hormesis.^30^ Hormesis phenomenon may be biphasic dose–response or a J-shaped or an inverted U-shaped curve.^31^ Extensive documentation showed that the “hormetic zones” for toxic agents have been established, and dysregulation of homeostasis is involved in a wide range of unexpected stresses or diseases.^32^ Of particular importance in the assessment of toxic agents, PCBs are recognized as very heterogeneous and depends on various factors such as the congeners involved, cell-types affected, the degree of exposure, and genetic predisposition.^33^ Polychlorinated biphenyls have been detected in wildlife, laboratory animals, and humans and often found to exhibit biphasic hormetic dose–responses^34^ and activate adaptive cellular stress pathways.^35^ In the current study, we provide direct evidence to indicate that hormetic effects in HeLa cell and the related signaling pathways due to exposure of 2 indicator congeners of PCBs (PCB150 and PCB180).

The results indicated that the effect of PCB150 and PCB180 on HeLa cell proliferation was concentration and time-dependent. The MTT assay confirmed the slight stimulation at 10^−3^ and 10^−2^ µg/mL for different time and inhibition at 10 and 15 µg/mL for 24 and 48 hours. Several studies have supported such theories of cell stimulation at low doses of noncoplanar PCBs in various cell models including human lung fibroblast cell (HELF), Vero cell, human breast cancer cell, and MCF-7 cell.^34^ Consistently, it was proven in this study that PCB150 and PCB180 induced a typical biphasic dose–response in HeLa cell which was in line with the typical character of hormesis.^36^

Generation of ROS is an effector mechanism to regulate cell growth, differentiation, and cell death.^37^ For several decades, efficient ROS formation has been shown to cause effective cell disruption and may eventually lead to cell death.^38^ However, more recently, less efficient ROS forming seem to be protective by triggering defense mechanisms that prevent cellular damage. This apparent paradox implies that different concentrations of ROS may evoke hormesis curve response.^39^ Here, we intended to demonstrate that our results were in keeping with hormetic relationship and directly linked to increased ROS production which induced endogenous oxidative damage in cell over time. In general, PCB150 exposed cells expressed more profound intracellular ROS levels as compared to PCB180. Therefore, the cells at low dose 10^−3^ µg/mL for 12 and 24 hours were not enough to cause profound oxidative stress and excessive influx of ROS at high dose 10^−2^, 1, 10, and 15 µg/mL approximately reaching the peak at 48 and 72 hours indicated cell signaling–mediated hormetic mechanisms. Taken together, our study is in accordance with previous studies that highlighted predominantly expressed ROS levels abolishes GSH which inevitably leads to compromised DNA function and integrity.^34^

Conversely, an emerging evidence has suggested that an upstream level of ROS is a transient phase which does not persist for an unlimited period of time, consequently it generates an innate endogenous expression of antioxidant enzymes and other stress defense pathways that enables ROS reduction.^40^ Different scholars believed that antioxidant enzyme activity increased in PCBs exposed cells under oxidative stress.^41^ The results presented here for PCB150 and PCB180 imply a novel mechanism by which the burden of ROS production is largely counteracted by intricate enzymatic scavengers for maintaining homeostasis and confer resistance to metabolic stress. In this experiment, higher antioxidant enzyme activities for MDA and SOD were maintained with high intensity of dose of PCB150 and PCB180 likely has a role to insure against an increase in ROS indicating a hormetic compensatory respond by the HeLa cells. Recent publications also support that the synthesis of SOD, which considered as a reliable biomarker of ROS contents, increases to reestablish the homeostasis in vivo.^42^ The contents of GSH-Px and MDA can reflect the level of oxidative damage in cells.^43^ Another mechanism to assess intracellular redox state is expression of GSH-Px, these data exhibited the down expression of GSH-Px in HeLa cells exposed to PCB150 and PCB180. Probably, the cause of this result is that an intensity of 15 µg/mL at different time was strong enough to cause toxic effects by oxidative stress. Our findings are in agreement with the previous work of Hashmi et al^34^ in which he demonstrated that decline in GSH activities appears to be induced cytotoxicity mediated through oxidative stress in HELF cells.

There is compelling evidence that genomic instability plays a prominent role in the initiation of carcinogenesis and it has also been linked to a variety of adverse health conditions.^44^ Hence, the ability to measure both endogenous levels of DNA damage and genotoxicant-induced DNA damage is particularly important. Diverse methods for measuring genomic damage have been developed including alkaline unwinding,^45^ DNA fiber analysis,^46^ direct-damage microscopy,^47^ and long amplification polymerase chain reaction.^48^ Among them, SCGE, also known as the comet assay, has also been used to measure DNA damage in cells or whole organisms for over 30 years.^49^ Widely embraced in toxicology and molecular biology, the technique can be used to measure DNA damage in mammalian tissues and cell culture models. This assay showed a positive response with PCB52 and PCB77 in human lymphocytes,^50^ PCB101 in HELF cells,^34^ PCB101 and PCB118 in fish cells in vitro,^51^ and PCB52 and PCB77 in human MDA-MB-231 (MDA) and MCF-7 breast cancer cells.^52^ The observations through this work were in line with previous studies, and our findings addressed the possible involvement of DNA damage in HeLa cell under PCB150 and PCB180 stress. The morphological observation revealed that comet length percentage detected for PCB150 increased in concentration-dependent manner and comet length percentage for PCB180 could be vividly seen suggesting HeLa cell is more sensitive to DNA damage for PCB 180 in comparison with PCB 150 (Figure 4II-A and B). Hence, it is speculated that PCB150 and PCB180 at high-intensity dose exposure (t3) induced DNA damage thus disrupting cell homeostasis which in turn reduce the capacity of the HeLa cell to withstand more severe insults.

There are reports that the well-known subgroups of MAPKs are linked in MAPK cascade in response to various stress signals including growth factors, hormones, cytokines, genotoxic, and oxidative stressors.^53^ It involves a sequence of functioning kinases, including ERK protein, which is known for its cell growth regulatory function, while JNK protein is known for primary regulation in apoptosis, inflammation, and the differentiation process.^54^ In the present study, the results indicated that a negligible phosphorylation of JNK, p-JNK, ERK1/2, p-ERK1/2, and actin at low concentration range (10^−3^ and 10^−2^ µg/mL) and phosphorylation increased at high dose (1, 10, and 15 µg/mL) within 12 hours compared to the control in PCB150 and PCB180 exposed HeLa cells, raising the possibility that compensatory mechanisms may be involved in maintaining the integrity of homeostasis. However, the time-dependent increase in the phosphorylation of apoptotic regulatory proteins could be significantly abolished the proliferation-promoting effect of antioxidant enzymes and induced cell death by PCB150 and PCB180. This was in line with the previous reports that MAPK activation has been implicated by ROS-mediated oxidative stress^55^ and induced cell death in PCBs exposed rat hepatocyte cell,^56^ mussel hemocytes,^57^ human MDA-MB-231 (MDA),^58^ human kidney cells (HK2),^52^ MCF-7 breast cancer cells,^59^ and HELF cells.^34^ Interestingly, this result is supporting our hypothesis that MAPK cascade is involved in the adaptive response of low doses and inhibitory response of high doses of PCBs on HeLa cells which commonly displays hormesis.

## Conclusion

This study demonstrated novel mechanism of PCB150 and PCB180 toxicity in HeLa cells which witnessed an expanding recognition of a β-shaped dose–response curve. For each congener, we were able to estimate its integrated effects and multiple interacting receptor/signaling pathways. Therefore, we conclude that PCB150 and PCB180 exhibited stimulatory effects at low doses and inhibitory effects at high doses on HeLa cell proliferation. Furthermore, the ROS production and antioxidant enzymes evoked in dose- and time-dependent manners. This study also suggests a model where intoxications to NDL PCBs impair the intracellular cascade by involving ERK and JNK phosphorylation and activity. This increase in activity may be due to elevation of systemic oxidative stress. It is worth mentioning that our estimates are in good agreement with previous exposure assessments and underlying cellular mechanisms providing fundamental insights into the risk assessment implications of hormesis in HeLa cells.
